# Key traits influencing the resistance of *Eucalyptus camaldulensis* to wind damage in coastal areas of South China

**DOI:** 10.3389/fpls.2024.1433670

**Published:** 2024-08-19

**Authors:** Xiuhua Shang, Peijian Zhang, Xiaoming Li, Youshuang Wang, Zhihua Wu

**Affiliations:** ^1^ Research Institute of Fast-growing Trees, Chinese Academy of Forestry, Zhanjiang, China; ^2^ State Key Laboratory of Efficient Production of Forest Resources, Beijing, China

**Keywords:** *Eucalyptus camaldulensis*, wind resistance, key traits, tree-pulling test, statistical analysis, analysis model

## Abstract

**Aims:**

China is one of the countries in the world most seriously affected by typhoons, which pose a great threat to the eucalyptus plantation industry. However, few studies have comprehensively accounted for the impact of key traits on the wind damage/resistance of eucalyptus.

**Methods:**

To identify the key factors affecting the wind resistance of eucalyptus, 20 eucalyptus genotypes were selected; a total of 18 traits, including the wind damage index, growth traits, and wood traits, were measured, and the wind resistance was determined via the tree-pulling test.

**Results:**

Correlation, principal component, canonical correlation, and path analyses were performed to evaluate these traits. Correlation analysis revealed that the wind resistance of eucalyptus plants was related to the tree height, volume, and duration of stress wave propagation. Principal components and tree-pulling variables were further used for correlation and path analyses. Canonical correlation analysis and the PA-OV model showed that holocellulose and lignin contents and fiber width, as well as growth traits, were important factors affecting the stability of standing trees under typhoon conditions. The key traits influencing the wind resistance of *Eucalyptu*s *camaldulensis*, which may provide a reference for evaluating the wind resistance of *Eucalyptus* varieties for forest management, were identified.

**Conclusion:**

This study provides a knowledge base for forest management and planning in typhoon-prone coastal areas, and provides a theoretical basis for the breeding and genetically improving eucalyptus stocks based on wind resistance characteristics.

## 1 Introduction

Wind is one of the most powerful environmental elements and a natural disturbance to forests (Mitchell, 2013). Wind can uproot, crack, split, or break tree trunks, branches, and limbs and thus cause serious damage to trees. Trees in many parts of the world die from wind damage every year, resulting in considerable economic and habitat losses (Quine et al., 1995; Schelhaas et al., 2003; Schütz et al., 2006). In the context of climate change, wind damage to trees and forests may become more frequent as the intensity of low-pressure systems outside or in tropical areas (hurricanes or typhoons) increases (Csilléry et al., 2017). Wind throws and snaps are the most common and serious wind disasters and not only threaten forest productivity but are also potential factors limiting tree height and forest carbon storage (Niklas, 2007; Bonnesoeur et al., 2016; Coomes et al., 2018). Therefore, it is necessary to understand the process of wind damage and its impacts on trees to reduce it for effective forest management.

The mechanical stability of trees, specifically the soil-root anchorage strength and stem strength, can be quantified under static loading by determining the maximum resistive turning moments at the stem and the base of the stem (Peltola et al., 2010). Insufficient soil-root anchorage causes tree failure by either uprooting or stem breakage (Nicoll and Ray, 1996). The influence of wind on trees and stands mainly depends on wind power and tree stability (Konôpka et al., 2016). If the wind exceeds the resistance of the trunk or root/soil systems, trees will break or become uprooted. The wind resistance of tree species is influenced by a combination of internal and external factors, including climate, topography, soil, standing wood characteristics, and forest management interventions (Nielsen, 1995; Ruel et al., 2000; Mitchell, 2013). For example, stand and site characteristics influence the wind resistance of stands (Peltola et al., 1999; Zubizarreta Gerendiain et al., 2016). The wind resistance of trees or stands also varies with age (Lekes and Dandul, 2000; Kuboyama et al., 2003). Tree diameter and species type significantly impact the probability of trunk breakage or uprooting in severe storms (Peterson, 2007; Nolet et al., 2012). Soil physical properties determine root morphology, overall size, and soil-root block (root ball) shape, which are the most important determinants of tree root anchoring in forests, and the interaction between roots and soil significantly affects tree trunk responses to wind (Nicoll et al., 2006; Waldron et al., 2013). As a complex tree structure system, the swing state of tree crowns under wind loading has a greater impact on tree trunks than does wind speed and direction (Spatz et al., 2007). Wood properties are also important factors influencing the wind resistance of trees (Putz et al., 1983; Xu et al., 2014). The wood density and the physical and mechanical properties of trunks influence the response of trees to wind loading (Gardiner et al., 2008). Trees with a high wood base density and low microfibril angle (MFA) showed greater wind resistance (Huang et al., 2017; Zanuncio et al., 2017). A high basic density of wood results in a greater mass of wood material per unit volume and confers stronger crushing resistance (Niklas and Spatz, 2012). However, Read et al (Read et al., 2011). reported that reducing wood density does not necessarily reduce wind resistance, which is affected by other characteristics, including cell-level characteristics such as the microfibril angle of the wood cell. Wind and trees have complex interactions that are influenced by several factors. Virot et al. (2016) argued that a wind speed of approximately 42 m/s can break over 50% of all trees in a forest, regardless of their characteristics. Therefore, the key traits/factors influencing the resistance of trees to wind damage for implementing effective forestry production and management strategies remain to be identified.

Eucalyptus is an important tree species in forest plantations and management in southern China due to its rapid growth, high yield, short rotation, and high economic value (Qi, 2002; Grattapaglia, 2008; Arnold et al., 2013; Xie et al., 2017). Tropical cyclones in the western North Pacific Ocean affect the coastal areas of southern China. Typhoons, strong storms that form in the Pacific, are frequent in the summer and greatly impact forestry in China’s coastal areas (Sun et al., 2010; Hong et al., 2012). It is important to assess the vulnerability of large-scale eucalyptus plantations to climate change (Booth, 2013). In particular, the damage caused by typhoons can restrict the production of eucalyptus wood (Braz et al., 2014; Boschetti et al., 2015). Wind can bend or break trees, reducing wood production and affecting the wood supply chain. In bent trees, apical dominance is reduced (Panshin and Zeeuw, 1981), while tree breakage increases production costs and efficiency, necessitating the cultivation of new small-diameter trees (Spinelli et al., 2009). Several studies conducted in China have indicated that wind damage predominantly occurs in 1- to 3-year-old eucalyptus plantations (Zhu, 2006; Ni et al., 2021). Similarly, in Brazil, wind was reported to cause damage to eucalyptus plantations primarily between 24 and 36 months after planting (Zanuncio et al., 2017). These findings highlight the vulnerability of young eucalyptus trees to wind-related damage. In recent years, few studies have investigated the wind resistance mechanism of eucalyptus plants at the individual tree level, especially the occurrence of stem breakage. Therefore, it is essential to investigate the resistance of eucalyptus plants to stem breakage during its early growth stages. In this study, to identify the key traits affecting the wind resistance of eucalyptus plants affected by stem/branch breakage, 20 genotypes of *E. camaldulensis* were selected, and the traits linked to the ability of the trees to withstand winds were evaluated via a new wind damage detection method. This study explains the factors that contribute to wind damage to eucalyptus trees, and the findings provide insights into reducing the risks in the management of commercial forests in coastal areas.

## 2 Materials and methods

### 2.1 Experimental materials

Sixty genotypes were collected from 40-month-old, open-pollinated progeny trials of *E. camaldulensis* at the South China Experiment Nursery (21.263°N, 110.098°E), located in Suixi, Zhanjiang, Guangdong Province, China. The experimental trial was planted in August 2012, with a row spacing of 2 m × 3 m, and laid out in a randomized complete block design (RCBD) with four replicates/blocks. Since planting, the experimental forest stand has been affected by five typhoons (Appendix A). After the fifth typhoon, 20 typical half-sib progenies (families), with three replicates per family, were selected for the tree-pulling test. Detailed information is provided in **Table 1**.

**Table 1:** Origin and code of the tested varieties of *E. camaldulensis* according to Shang et al. (2019).

No.	Family name^1^	Source of seedlot	Location^2^	Type^3^	Latitude	Longitude(E)	Altitude (m asl)	Mean annual rainfall(mm yr^-1^)
1	C04	India	DPK	CSO 1	–	–	–	–
2	C013	India	KUL	SSO 2	–	–	–	–
3	C014	India	DPK	CSO 1	–	–	–	–
4	C023	India	DPK	CSO 1	–	–	–	–
5	C033	India	DPK	CSO 1	–	–	–	–
6	C036	India	DPK	CSO 1	–	–	–	–
7	C046	India	MPM	CSO 1	–	–	–	–
8	C076	India	MPM	SSO 1	–	–	–	–
9	C079	India	MPM	SSO 1	–	–	–	–
10	C080	India	MPM	SSO 1	–	–	–	–
11	CA5	Australia	Laura River	NS	15°37’ S	144°31’	95	988
12	CA7	Australia	Laura River	NS	15°37’ S	144°31’	95	988
13	CA8	Australia	Laura River	NS	15°37’ S	144°31’	95	988
14	CA9	Australia	Kennedy River	NS	15°23’ S’	144°10’	80	988
15	CA16	Australia	Morehead River	NS	15°02’ S	143°40’	60	1201
16	CA21	Australia	Palmer River	NS	16°07’ S	144°48’	410	1041
17	CA22	Australia	Palmer River	NS	16°07’ S	144°48’	410	1041
18	CA26	Australia	Normanby Rivers Normanby Rivers	NS	15°46’ S	144°59’	205	954
19	CA27	Australia	Normanby Rivers	NS	15°46’ S	144°59’	205	954
20	CA28	Australia	Normanby Rivers	NS	15°46’ S	144°59’	205	954

^1^ Seedlots or family names commencing with C were supplied by CSIRO’s Australian Tree Seed Centre.

^2^ The exact locations of the C0 Indian seed orchards are uncertain. The three categories are as follows: CSO, clonal seed orchard; SSO, seedling seed orchard; and NS, natural stand.

^3^Type categories are as follows: SSO, seedling seed orchard; CSO, clonal seed orchard; NS, natural stand.

### 2.2 Measurement indices and methods

The growth traits of the forest stands, including tree height (H), diameter at breast height (DBH), and bark thickness (BT), were measured using a Vertex IV instrument (Haglof, Sweden), measuring tape, and Vernier caliper, respectively, and calculate the volume(VOL). A piece of bark approximately 2-4 cm in height at a height of 1.3 m was removed with a knife to measure the BT. The wind damage index (WD) was measured after the “rainbow typhoon” in October 2015 according to Wang et al (Wang et al., 2014), and post-event damage could be assessed subjectively using the following criteria: a score of 0 indicates a healthy tree with no obvious trunk inclination, a score of 1 denotes low damage, and the distance between trunk inclination and the vertical line was less than 30 degrees; a score of 2 signifies low to moderate damage, with the angle between trunk inclination and the vertical line being 30° to 60°; a score of 3 indicates moderate damage, with the angle of 60° to 90° between trunk inclination and the vertical line; a score of 4 denotes great damage, along with trunk lodging or uprooting; a score of 5 signifies serious damage, and the tree trunk or treetop was damaged or broken.

Pilodyn is a non-destructive testing instrument used to indirectly determine wood density. It can be used to effectively and indirectly evaluate wood properties such as strength and density (Luan et al., 2011). Pilodyn (Pilodyn penetration; PN) was measured with a Pilodyn 6J Forest device (Proceq Switzerland), with a pin diameter of 2.5 mm at the height of the living tree’s chest in the north−south direction. The living tree was not peeled, and data were obtained from both the south and north directions, followed by the calculation of the average of the two directions. The stress wave velocity of wood is directly related to its physical and mechanical properties, which can be determined by measuring the propagation time (T) of the stress wave or stress wave velocity. The time of stress wave propagation (FP) in standing trees was measured using FAKOPP 2D (FAKOPP Enterprise Bt, Hungary). The two probes were fixed on the trunk at an axial interval of 1.2 m, the coaxial connection cable (connector) was connected to the sensor, and then one of the probes was hit with a hammer; the time of the stress wave propagation through the other probe was displayed on the screen, and the average value of 5 replications was taken.

The wood core samples were used to determine the fiber morphological characteristics in the north−south direction at a height of 1.3 m for each tree using a tree growth cone developed by Haglof, Sweden, with an inner diameter of 4.3 mm. The wood core sample (3-5 g) was soaked in a 1:1 solution of H_2_O_2_:CH_3_COOH at 65°C for 10 h. A disperser was used to thoroughly disperse the solution for approximately 10 min, and the solution was then filtered through a 150-mesh detergent bag. Thereafter, a small amount of softened pulp was taken and put into a standard disperser containing high-purity water. The solution was manually stirred to disperse the pulp fibers evenly. An appropriate volume of the dispersed solution was taken for the test and poured into a special plastic measuring cup, followed by setting the count of measured fibers to 5000. According to the operating procedures of the LDA 02 Hi-Res Fiber Quality Analyzer (OPTEST, Canada), the fiber length (FL) and fiber width (FW) were measured, based on which the ratio of fiber length to fiber width (FLW) was calculated.

**Figure 1:**
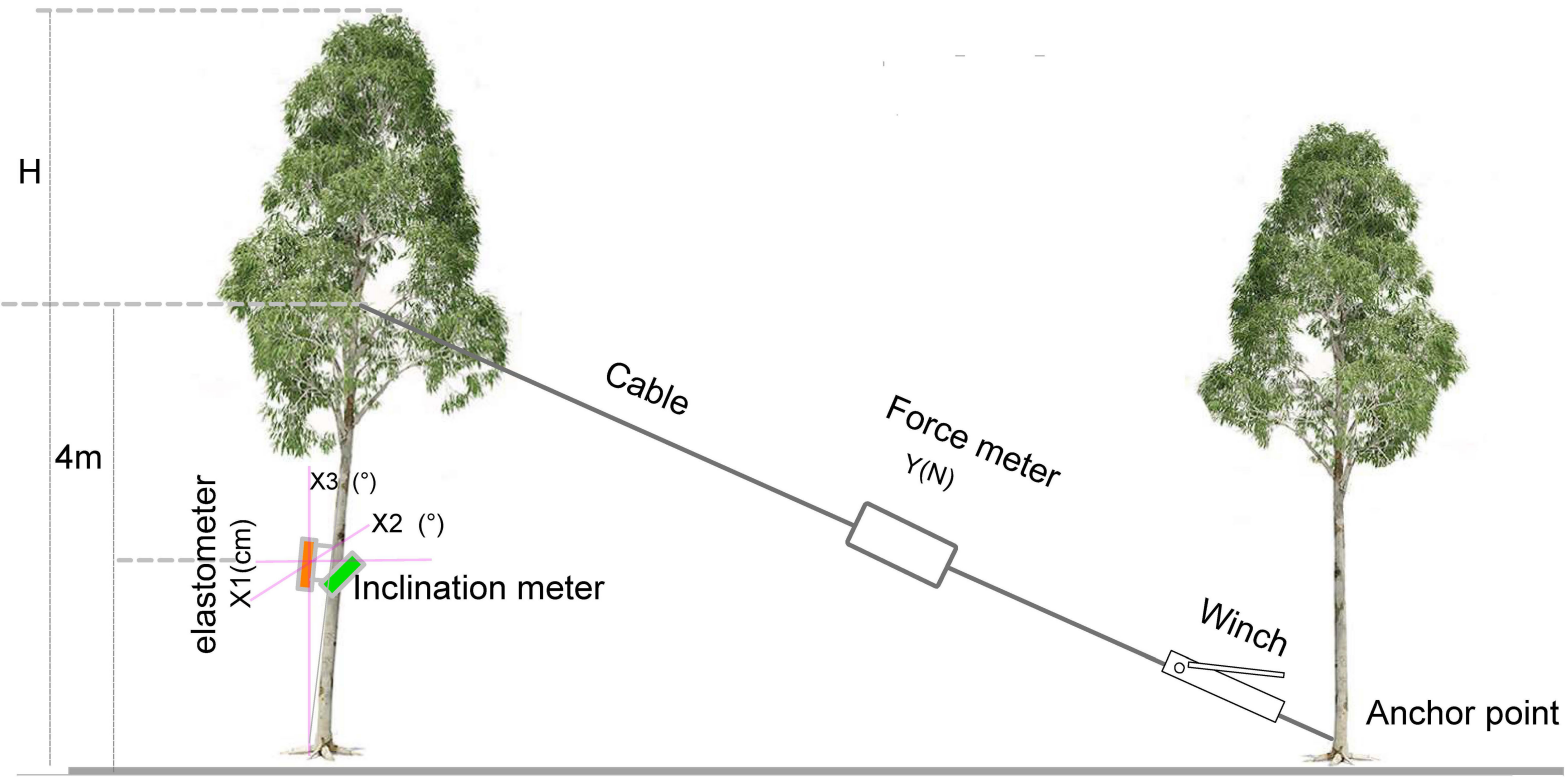
Schematic diagram of the simulation of wind damage to trees.

After completing the growth and non-destructive measurement of tree properties, 60 experimental trees were subjected to the tree-pulling test using the PiCUS TreeQinetic system (Argus electronic, Rostock, Germany) to simulate wind damage. Based on our tree-pulling tests, due to the softness of the wood of 40-month-old eucalyptus trees, pulling the tree from a greater height decreased only the proportions of the top part of the standing tree without causing significant trunk tilting or breakage. To assess the tensile capacity of standing trees to achieve a significant effect in the tree-pulling test, a tree height of 4 m was selected as an anchor point according to previous studies (Zanuncio et al., 2017; Krišāns et al., 2022) (**Figure 1**). Then, an elastomer and inclinometer were used to measure the elastic deformation and trunk inclination angle, respectively, at a height of 2 m. Simultaneously, the pulling force of the cable was recorded. When the trunk tilted 30 degrees at 4 m, the measurements of elastic deformation and inclination angle were recorded. The cable length from the pulling point to the anchor point and the horizontal distance between the base of the tested tree and the anchor point were also recorded. The recorded parameters of the tree-pulling test included the pulling force (Y_force_), which is the tension measured using a force meter; X1, the deformation degree of the trunk (µm) measured using an elastomer; X2, the inclination angle of the trunk perpendicular to the pulling direction (°) measured by the inclinometer; and X3, the trunk inclination angle along the pulling direction (°).

After performing the tree-pulling test, the sample trees were cut down. Following the felling of the sample wood, logs within the range of 1.3-3.3 and 5.3-7.3 m in trunk height were selected for the assessment of the wood’s physical and mechanical properties. When the wood moisture content reached approximately 12%, logs from the sample trees were prepared for the determination of the physical and mechanical properties of the wood according to Chinese national standard GB/T 1930∼1941–2009; Methods for Sawing and Sampling of Physical and Mechanical Test Pieces of Wood. Wood basic density (WBD) was measured by the drainage method according to the national standard “Method for Determination of Wood Density” (GB/T 1933-2009). The specimen dimensions were 20 mm × 20 mm × 20 mm (in parallel), and distilled water was added 1-2 cm above the sample surface. The sample was soaked to saturation, and the volume of each sample was measured based on the saturated water content; thereafter, the sample was placed in an oven at 103 ± 2°C until it was completely dry, and each sample was weighed. The bending strength (MOR) of the samples from different genotypes was determined according to the “Method of Testing in Bending Strength of Wood” (GB/T1936.1-2009). The specimen dimensions were 20 mm (radial) × 20 mm (chord) × 300 mm (longitudinal). The operation procedure was as follows: first, the specimen was symmetrically mounted on the two supports in the mechanical test machine, with a distance of 240 mm between the supports. In the central loading test of the specimen, the load was applied uniformly along the radial direction in the middle of the specimen at a loading speed of 10 mm/min. The bending modulus of elasticity (MOE) was determined according to the “Method for determination of the modulus of elasticity in static bending of wood” (GB/T1936.2-2009). The shear strength parallel to the grain (SSG) was determined by the “Method of testing in shearing strength parallel to the grain of the wood” (GB/T 1937-2009). The compressive strength parallel to the grain (CP) was determined by the “method of testing in compressive strength parallel to the grain of the wood” (GB/T 1935-2009). The MOR, MOE, SSG, and CP were measured with an Instron 5582 universal testing machine (Instron Corporation, USA).

The cellulose content (CC) was determined via iodometric titration of potassium dichromate. The sample was powdered (0.05-0.06 g) and placed into a centrifuge tube containing a mixture of 5 mL nitric acid and acetic acid, which was then boiled for 25 min. After the centrifugation of the mixture, 8 mL of sulfuric acid and 10 mL of 0.5-N potassium dichromate solution were added to dissolve the precipitate, and the solution was placed in a boiling water bath for 10 min. After cooling, 3 drops of ferrous reagent were added and titrated with 0.1-N Mohr’s salt solution. The hemicellulose content (HC) was measured using a combination of hydrochloric acid hydrolysis and the dinitro salicylic acid (DNS) method. Approximately 0.1-0.2 g of sample powder was taken into a beaker, and 15 mL of the 80% calcium nitrate solution was added; the mixture was then heated for 5 min, allowed to cool, and centrifuged. Thereafter, 10 mL of a 2 N hydrochloric acid solution was added to boiling water and heated for 45 min to completely hydrolyze hemicellulose, followed by centrifugation, the addition of phenolphthalein, and neutralization with a 2 N sodium hydroxide solution until the solution turned orange−red. Finally, 10 mL of the basic copper reagent was added, and the mixture was stirred and heated for 15 min. Then, 5 mL of the oxalate-sulfuric acid mixture and 0.5 mL of 5% starch were added, and the mixture was titrated with 0.01-N sodium thiosulfate solution. The Klason method was used to calculate the lignin content (LC). Approximately 0.05-0.1 g of the sample powder was taken into a centrifuge tube, soaked and washed in 10 mL of 1% acetic acid, and centrifuged. The mixture was then soaked in 3-4 mL of acetone for 3 min, allowed to precipitate and dry in boiling water, 3 mL of 73% sulfuric acid was added, and the mixture was allowed to stand for 16 h and then placed in boiling water for 5 min. After cooling, 0.5 mL of the 10% barium chloride solution was added and centrifuged, and finally, 10 mL of 0.5-N potassium dichromate solution and 8 mL of sulfuric acid were added and titrated with 0.1-N Mohr’s salt solution using ferro-methyl reagent as an external indicator. The procedure for each sample was repeated three times, and then, the average values were taken. The detailed operating instructions are provided by (Liang et al., 2020; Lu, 2022).

### 2.3 Statistical data analysis

Microsoft Excel 2010 (V2020-L.1207, CMGE, Beijing, China, 2021) was used for collection, collation, and preliminary data analysis. R Statistical Software (v 4.1.2, R Core Team, Vienna, Austria, 2021) and RStudio (v 1.1.463, RStudio Team, Vienna, Austria, 2021) were used to perform correlation analysis, principal component analysis (PCA), and canonical correlation analysis (CCA) of the data. Since data are a means of collecting measurements, standardized values were used for PCA and path analysis to reduce the dimensionality of different datasets.

## 3 Results

### 3.1 Analysis of standing tree traits

**Figure 2:**
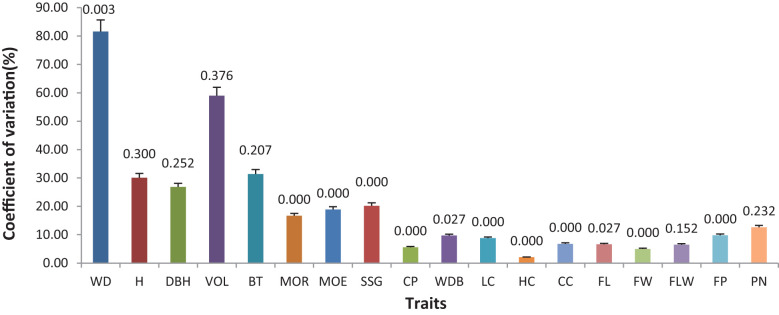
Coefficient of variation (CV) and analysis of variance (ANOVA) for different traits among different families: The height of each column represents the coefficient of variation for each trait, while the values on the column indicate the P value obtained from the ANOVA test for each trait.

The analysis of variance (ANOVA) results showed that there were differences in various traits among different families (**Figure 2**; **Table 2**). However, there were no significant differences in H, DBH, VOL, BT, FLW, or PN (P>0.05), while the differences in WBD were significant (P<0.05). Highly significant differences (P < 0.01) were detected in WD, MOR, MOE, SSG, CP, LC, HC, CC, FL, FW, and FP. The coefficient of variation (CV) for each trait varied among the different families. The coefficient of variation for WD was 81.60%, indicating a strong variation, and V, BT, and H also exhibited high coefficients of variation, all of which were greater than 30%. The CVs for traits related to fiber morphology (FL, FW, and FLW) and chemical composition (CC, HC, and LC) were less than 10%. The coefficients of variation for the physical and mechanical indicators (MOR, MOE, SSG, and CP) ranged from 5.60% to 20.02%.

**Table 2:** Result of the growth, wood properties and wind damage index among different families.

Family	WD	H(m)	DBH (cm)	VOL (m^-3^)/1000	BT (cm)	MOR (Mpa)	MOE (Mpa)	SSG (Mpa)	CP (Mpa)	WDB (g/cm^-3^)	LC (%)	HC (%)	CC (%)	FL (mm)	FW (μm)	FLW	FP (s)	PN (mm)
C04	2.71	11.77	8.99	38.38	0.58	81.05	7394	27.99	48.15	0.47	23.72	27.47	45.19	0.61	25.26	24.04	490.75	12.91
C013	2.88	11.36	11.66	36.82	0.61	71.16	6741	26.24	44.49	0.42	24.10	27.71	45.30	0.59	26.62	22.32	490.60	13.64
C014	2.20	8.97	11.38	46.52	0.73	68.72	6011	21.58	43.00	0.44	19.52	28.72	51.13	0.59	25.38	23.37	520.67	12.90
C023	1.56	11.41	10.93	45.66	0.71	76.71	7403	27.03	49.65	0.49	22.99	27.82	50.95	0.53	24.60	21.66	533.38	14.33
C033	1.50	9.15	9.95	38.43	0.58	73.53	8470	25.07	53.06	0.45	19.86	28.58	50.55	0.59	25.86	22.91	478.50	13.79
C036	2.60	7.43	7.60	31.75	0.32	71.79	7103	23.07	41.42	0.46	19.53	28.28	51.80	0.57	25.90	22.20	448.67	13.17
C046	1.00	11.24	9.81	42.07	0.67	58.00	5056	19.87	42.09	0.39	24.16	27.56	54.18	0.53	24.03	22.13	567.33	13.52
C076	1.25	11.56	9.27	41.61	0.50	78.54	7426	30.17	49.84	0.50	21.91	27.68	46.80	0.58	24.89	23.48	482.00	13.32
C079	2.50	8.13	9.48	26.58	0.55	76.54	7230	19.54	44.73	0.43	26.13	27.57	46.03	0.57	25.50	22.55	456.00	13.48
C080	1.08	11.48	11.37	42.98	0.65	63.66	6365	23.69	40.12	0.41	22.92	27.32	44.80	0.57	24.37	23.23	551.00	14.52
CA5	2.20	9.58	11.01	43.07	0.60	86.54	7813	30.04	46.42	0.50	21.47	28.84	48.34	0.59	26.02	22.51	459.70	11.80
CA7	1.95	9.95	10.46	49.05	0.58	82.99	7233	27.80	47.44	0.47	25.47	27.37	46.12	0.60	25.99	23.00	460.90	12.95
CA8	1.93	10.08	10.76	42.91	0.62	81.42	7346	27.16	48.61	0.49	24.58	27.15	43.98	0.57	24.77	22.96	488.73	12.59
CA9	1.60	12.03	11.17	51.45	0.65	58.69	5904	16.20	37.06	0.41	23.51	26.96	42.26	0.58	25.22	22.86	508.45	13.23
CA16	1.20	11.35	10.59	44.97	0.60	73.84	6638	27.41	43.45	0.44	19.37	27.93	46.59	0.58	25.37	22.70	510.64	13.80
CA21	1.00	11.67	12.13	55.34	0.70	83.94	7231	23.32	47.76	0.47	22.47	28.13	49.19	0.59	25.53	22.95	503.60	13.57
CA22	1.25	10.65	9.50	40.97	0.57	89.00	8165	31.25	52.41	0.50	21.96	28.62	51.64	0.58	25.28	22.88	489.17	12.53
CA26	1.71	9.92	11.24	45.72	0.61	78.21	7616	29.79	49.94	0.49	24.79	28.98	49.93	0.60	25.53	23.58	461.71	12.84
CA27	1.10	10.35	9.53	41.67	0.56	84.77	7705	24.26	49.02	0.51	24.18	28.48	42.43	0.59	26.23	22.39	500.30	13.00
CA28	2.38	9.89	8.97	33.75	0.48	84.86	7943	29.24	50.55	0.49	23.40	27.57	48.17	0.57	24.36	23.51	505.80	12.20

### 3.2 Correlation analysis of each trait

**Figure 3:**
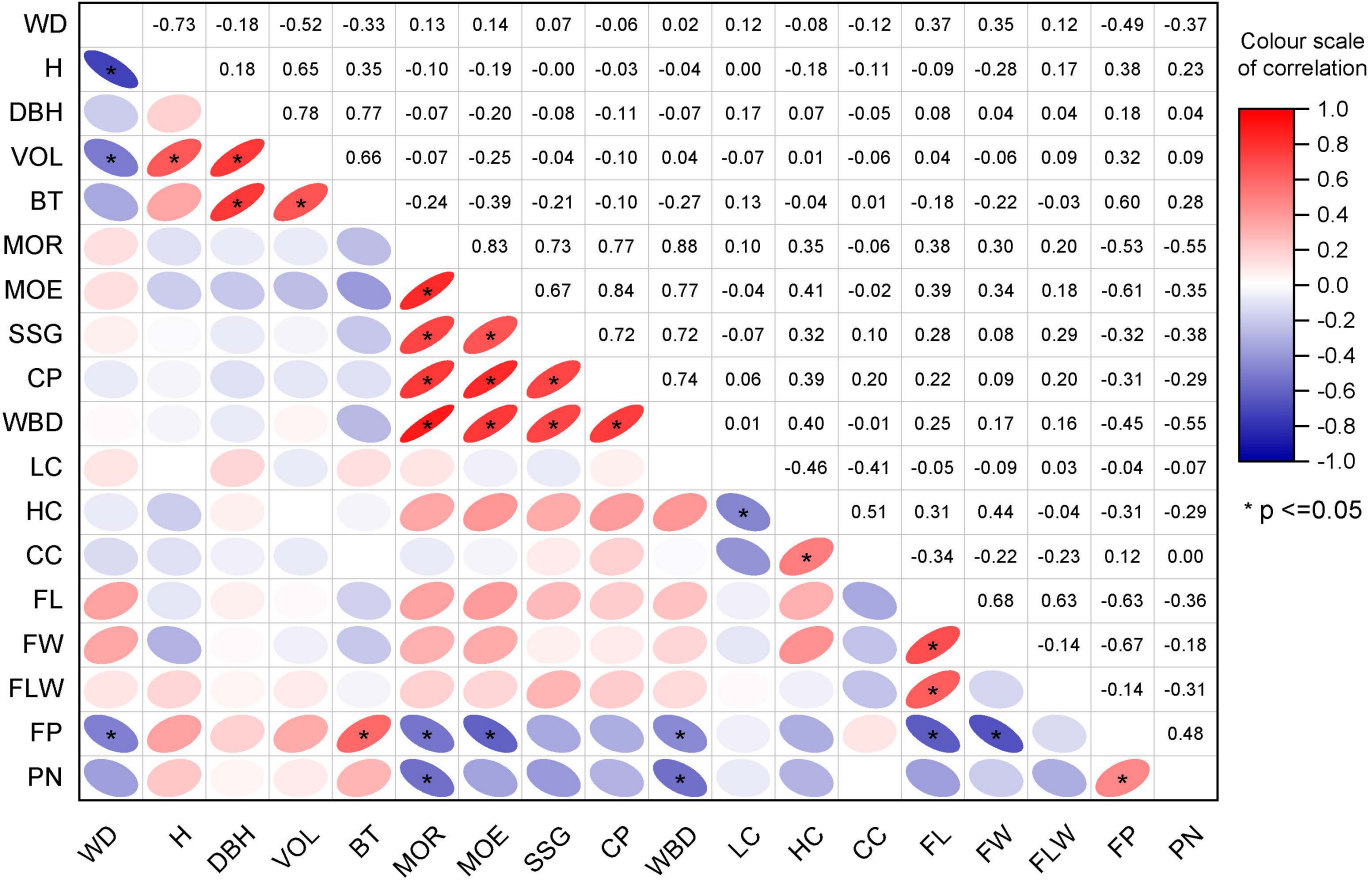
The correlation matrix between traits: The value in each cell in the figure represents the correlation coefficient between two traits. The higher the relative value of the correlation coefficient is, the darker the color. Red indicates a positive correlation, while purple denotes a negative correlation. * indicate significant difference at 0.05.

The correlation matrix shows that the clustering was based on the degree of correlation between different traits (**Figure 3**). Growth traits such as H, DBH, VOL, and BT were clustered in one group, while non-destructive testing properties, including FP and PN, were clustered in one group, and physical and mechanical properties such as MOE, SSR, and CP formed one cluster; fiber morphological variables and contents of cell wall components (LC, HC, CC, and FL), as well as other wood properties, including FW and FLW, were in one cluster. There was a strong correlation among the variables within the same category. For example, the correlation coefficient between wood properties was significant and greater than 0.6. Similar trends were observed for the non-destructive testing properties of the wood and wood fiber properties WD, which showed a very strong correlation with H, VOL, and PN.

### 3.3 Principal component analysis of traits

**Figure 4:**
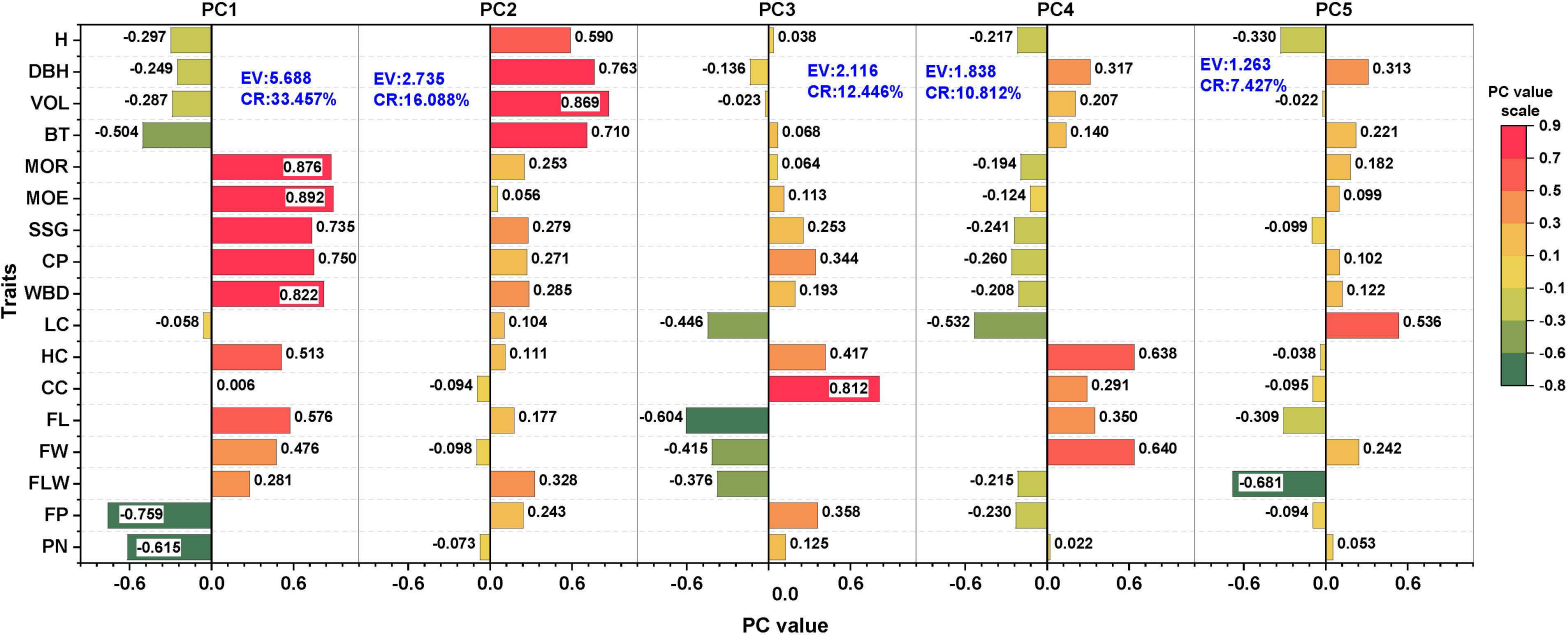
Eigenvectors and percentages of the accumulated contribution of principal components (PCs); CR, Contribution rate (%) and EV, Eigenvalue.

According to the PCA of 17 traits except for WD (**Figure 4**), the contribution rates of the first five principal components (PCs) were 33.457%, 16.088%, 12.446%, 10.812%, and 7.427%, respectively, with a cumulative contribution rate of 80.230%. The five PCs explained more than 80% of the total variation, and therefore, the 17 investigated traits were divided into five new independent sets of comprehensive indices. PC1 had a strong positive correlation with the MOE, MOR, WBD, CP, and SSG, which mainly reflected the physical and mechanical properties of the standing wood, with an eigenvalue of 5.688. PC2 had the strongest positive correlation with VOL, DBH, BT, and H, which mainly reflected the growth status of the standing wood, with an eigenvalue of 2.735. PC3 had a strong correlation with CC and FL, with an eigenvalue of 2.116. PC4, however, was positively correlated with HC, FW, LC, and FL, which are the main traits of wood fiber. PC5 was closely correlated with FLW and LC, which are also wood fiber variables. PC3, PC4, and PC5 were mainly related to the chemical composition and fiber morphological traits of the standing wood.

### 3.4 Tree-pulling test of standing trees

**Figure 5:**
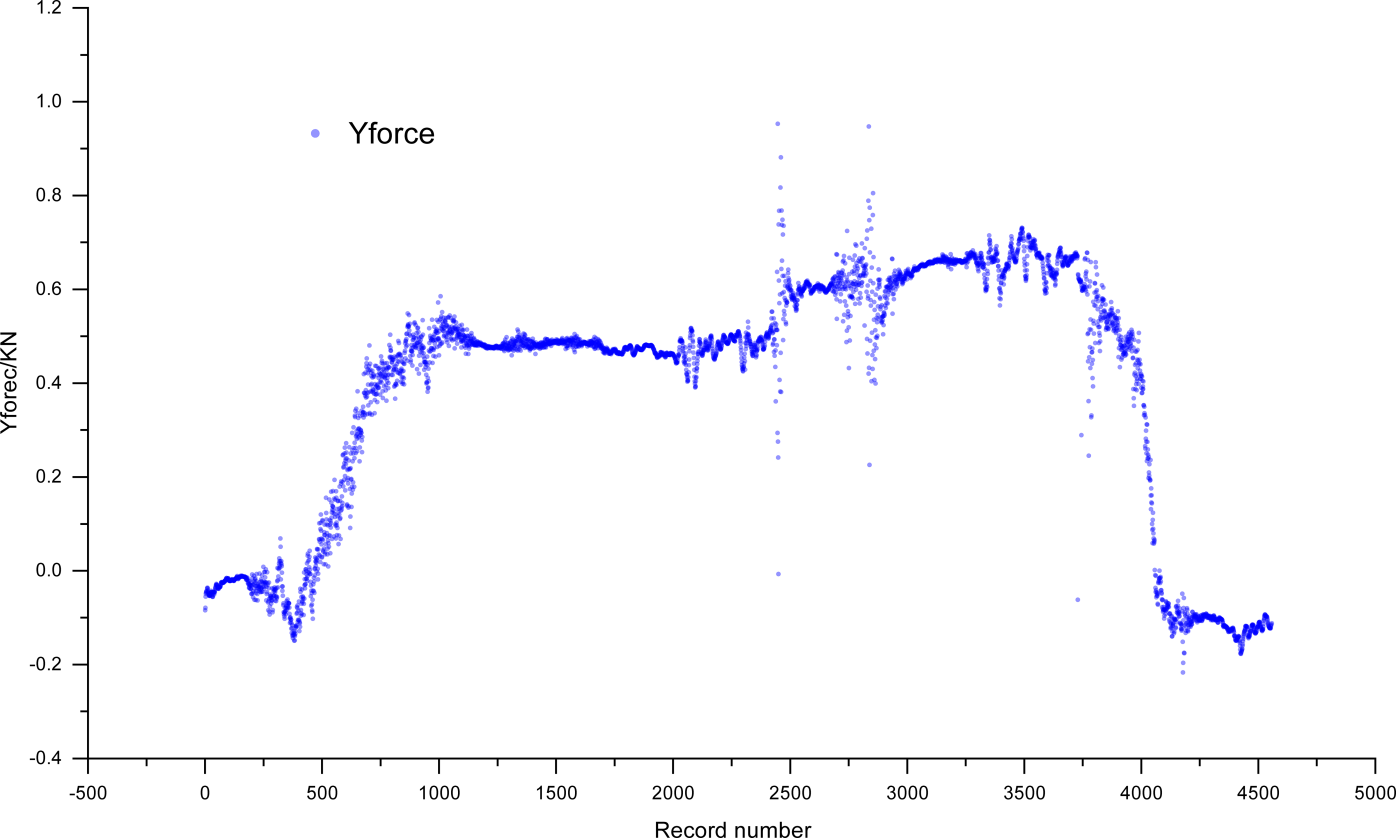
The tension of standing trees of the C033 family of *eucalyptus camaldulensis* during the tree-pulling test.

The wind damage indices of 60 standing trees were obtained by simulating wind damage via a tree-pulling test. Taking C033 as an object, the tensile force of upright trees was determined when the standing tree was pulled and then released during the whole period of the tree-pulling test (**Figure 5**). Since the tested trunk was a bioelastic body and wind disturbance to the crown occurred, the tree experienced a certain level of vibration, leading to a narrow range of variation in the pulling force. In general, the increase in the pulling force showed a linear relationship with the elastic deformation (X1) and inclination angle (X2 and X3) of the trunk.

The variables were obtained using a tree-pulling test, and their fitted models were developed using DataFit 9.0 (Oakdale Engineering, Oakdale, PA). The datasets Y_force_, X1, X2, and X3 obtained from the tree-pulling tests for each plant, exceed 4,000, as depicted in **Figure 1** (CO33). Regression equations were developed using the datasets X1, X2, and X3, corresponding to over 12,000 Y_forces_ from various pulling conditions. A significant correlation was observed between Y_force_ and X1, X2, and X3 (**Table 3**). Therefore, the fitting regression equation can be established with Y_force_, X1, X2, and X3 as follows: *y = aX1 + bX2 + cX3 + d*. The *R* ^2^ values of the regression equations for *E. camaldulensis* ranged from 0.6371 to 0.9673 and reached highly significant levels (P < 0.01) for all fitting equations. These results showed that the equations could accurately reflect the dynamic relationship between Y_force_ and X1, X2, and X3. The greater the coefficient for the fitting equation (“a”), the greater the Y_force_ of standing trees at the same deformation degree. The greater the coefficient for the fitting equation (“b”), the greater the pulling Y_force_ at the same vertical inclination angle of the standing stand and under the same pulling force direction. Furthermore, the larger the value of parameter “c” of the fitting equation is, the greater the pulling force of the standing tree at the inclination angle under the same pulling force direction.

**Table 3:** The regression equations and regression statistics of pull trees and three factors in the pull tree simulation wind damage tree test.

Family	Fitted equation	a	b	c	d	Residual error	R^2^	DF	SS	MS	F value
CA26	Y=aX1+bX2+cX3+d	-0.0003	-0.1739	0.0492	-0.1720	0.1028	0.9050	3	847.75	282.58	26753.22
CA21	Y=aX1+bX2+cX3+d	0.0000	0.1172	0.0433	0.0164	0.0744	0.8960	3	95.54	31.85	5744.26
CA22	Y=aX1+bX2+cX3+d	-0.0004	-0.1496	0.0656	-0.0980	0.1066	0.8799	3	316.43	105.48	9283.49
CA28	Y=aX1+bX2+cX3+d	-0.0000	0.1260	-0.0742	0.0027	0.1935	0.7937	3	624.47	208.16	5558.42
CO46	Y=aX1+bX2+cX3+d	-0.0005	0.0038	-0.0342	0.2110	0.1221	0.6371	3	106.51	35.50	2380.32
C076	Y=aX1+bX2+cX3+d	-0.0002	-0.2320	0.0976	-0.0517	0.1757	0.8977	3	1295.41	431.80	13990.91
C079	Y=aX1+bX2+cX3+d	-0.0002	0.2355	0.0865	0.0013	0.1012	0.9600	3	1301.67	433.89	42330.83
C05	Y=aX1+bX2+cX3+d	-0.0007	-0.2068	0.1115	0.0601	0.0736	0.8983	3	221.38	73.79	12659.10
CA27	Y=aX1+bX2+cX3+d	-0.0003	0.0020	-0.0011	0.0115	0.1019	0.8431	3	184.66	61.55	5923.75
C013	Y=aX1+bX2+cX3+d	-0.0003	-0.0114	0.0140	0.6598	0.3941	0.8472	3	610.34	203.45	1309.79
C033	Y=aX1+bX2+cX3+d	-0.0011	0.3382	0.7866	-0.0618	0.1284	0.8396	3	405.45	135.15	8190.75
C04	Y=aX1+bX2+cX3+d	-0.0006	-0.0075	-0.0128	-0.0142	0.1013	0.9110	3	262.88	87.63	8542.76
C036	Y=aX1+bX2+cX3+d	-0.0003	0.0028	0.0588	-0.0133	0.1151	0.9120	3	731.80	243.94	18421.70
CA9	Y=aX1+bX2+cX3+d	-0.0010	0.4044	-0.0487	0.1034	0.1390	0.9673	3	1618.50	539.50	27926.84
CA16	Y=aX1+bX2+cX3+d	0.00005	-0.0241	0.1146	-0.0058	0.1780	0.7077	3	259.10	86.67	2727.01
C023	Y=aX1+bX2+cX3+d	-0.0002	-0.2123	0.0994	0.0188	0.0982	0.9737	3	3309.73	1103.24	114199.56
CA8	Y=aX1+bX2+cX3+d	-0.0003	-0.04238	0.0564	0.0264	0.1347	0.9158	3	2668.23	889.41	49009.53
CA7	Y=aX1+bX2+cX3+d	0.0016	-0.0546	0.03386	-0.0204	0.0852	0.9322	3	112.88	37.63	5187.85
C014	Y=aX1+bX2+cX3+d	-0.0005	0.0999	0.0053	-0.0013	0.0670	0.9325	3	434.90	144.97	32238.10
CO80	Y=aX1+bX2+cX3+d	-0.0019	0.1972	-0.0247	0.0038	0.0876	0.8848	3	338.73	112.91	14699.96

Y represents the pulling force in a fitting equation (N), whereas X1 indicates the degree of deformation of the tree in a fitting equation (cm); X2 denotes the standing and pulling direction perpendicular to the tilt angle of trees measured by the inclinometer (°), while X3 represents the tensile force in the direction of the tilt angle of trees measured by the inclinometer (°).

### 3.5 Canonical correlation analysis

**Figure 6:**
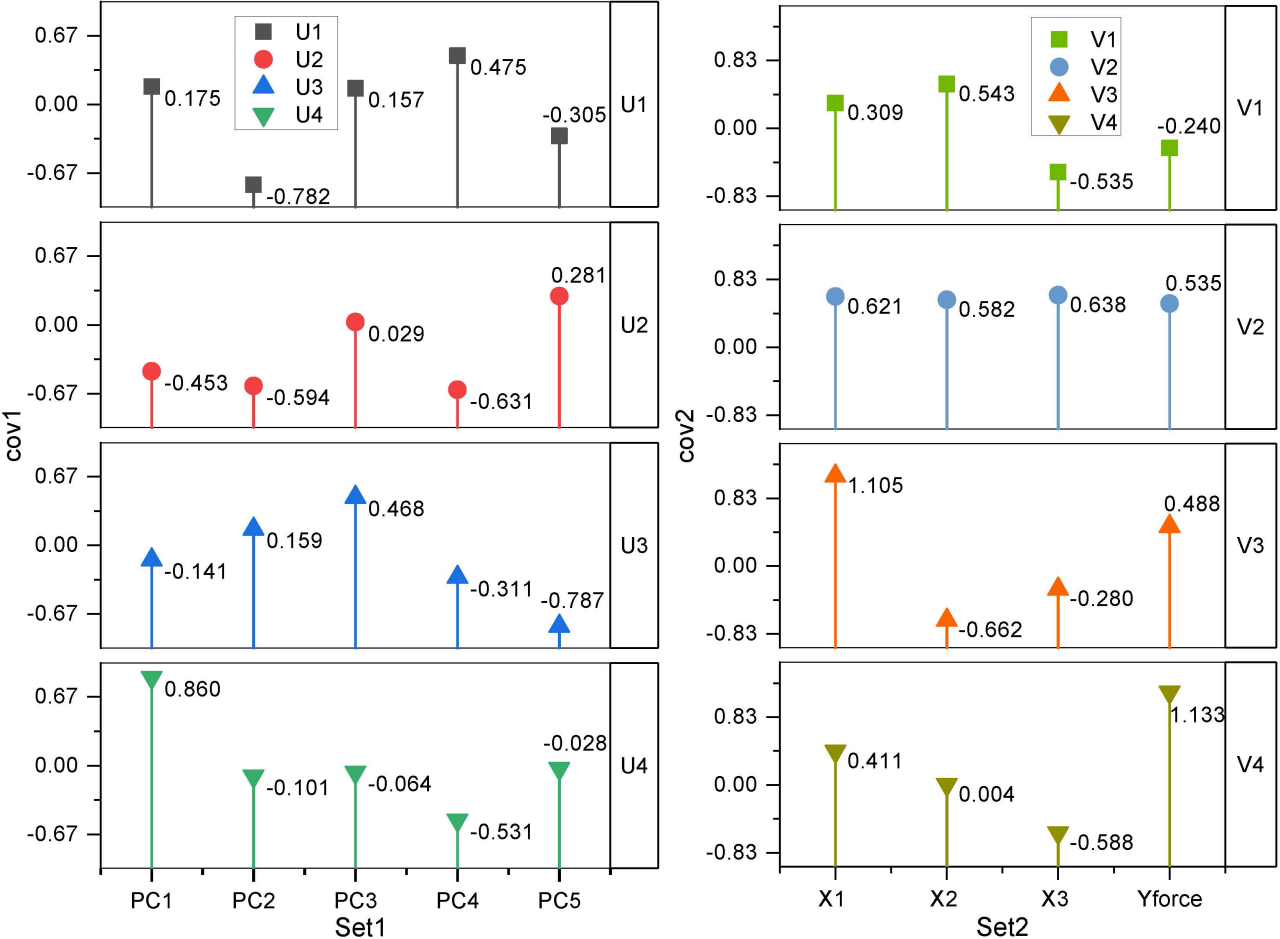
Principal components (PCs) of eucalyptus traits and coefficients for canonical variables in the tree-pulling test based on canonical correlation analysis.

Among the 13 wood traits, only FP was related to wind resistance, and thus, the correlation analysis between single-factor variables could not reveal the real cause of wind resistance in the forest. Canonical correlation analysis (CCA) was performed between the first five principal components (PC1-PC5) obtained from the PCA of traits related to wind resistance and the four variables, Y_force_, X1, X2, and X3, obtained from wind damage simulated by the tree-pulling test to identify the key traits affecting the wind resistance of trees. As shown in **Table 4**, the first two canonical correlation coefficients were 0.9547 and 0.9012, respectively, and were strongly correlated at highly significant levels (P < 0.01). The first two pairs of canonical covariates were used to examine the relationship between the set of variables of the standing tree in the tree-pulling test (**Figure 6**). For eucalyptus variables, the first covariate (U1) in set 1 of the data was strongly affected by PC2 (-0.7820) and PC4 (0.4747), and the second covariate U2 was similarly strongly affected by PC4 (-0.6311) and PC2 (-0.5936). The coefficients of the first group of typical variables mainly reflected the positive correlation between PC2 and X1 and the negative correlation between PC2 and X3, while the coefficients of the second group of typical variables mainly reflected the positive correlation between PC4 and X1 and X3. Therefore, PC2 and PC4 were the main factors affecting the wind resistance of eucalyptus.

**Table 4:** Statistical analysis of the canonical correlations.

Dimension	Correlation coefficient	Wilk’s	F	Chi-square value	Df	P value
1	0.9547	0.0116	5.3046	37.4328	20	5.7641×10^-6^
2	0.9012	0.1310	3.0857	32.0405	12	5.3751×10^-3^
3	0.5446	0.6979	0.8536	26.0000	6	0.5411
4	0.0883	0.9922	0.0550	14.0000	2	0.9467

### 3.6 Estimated path analysis using the PA-OV model

**Figure 7:**
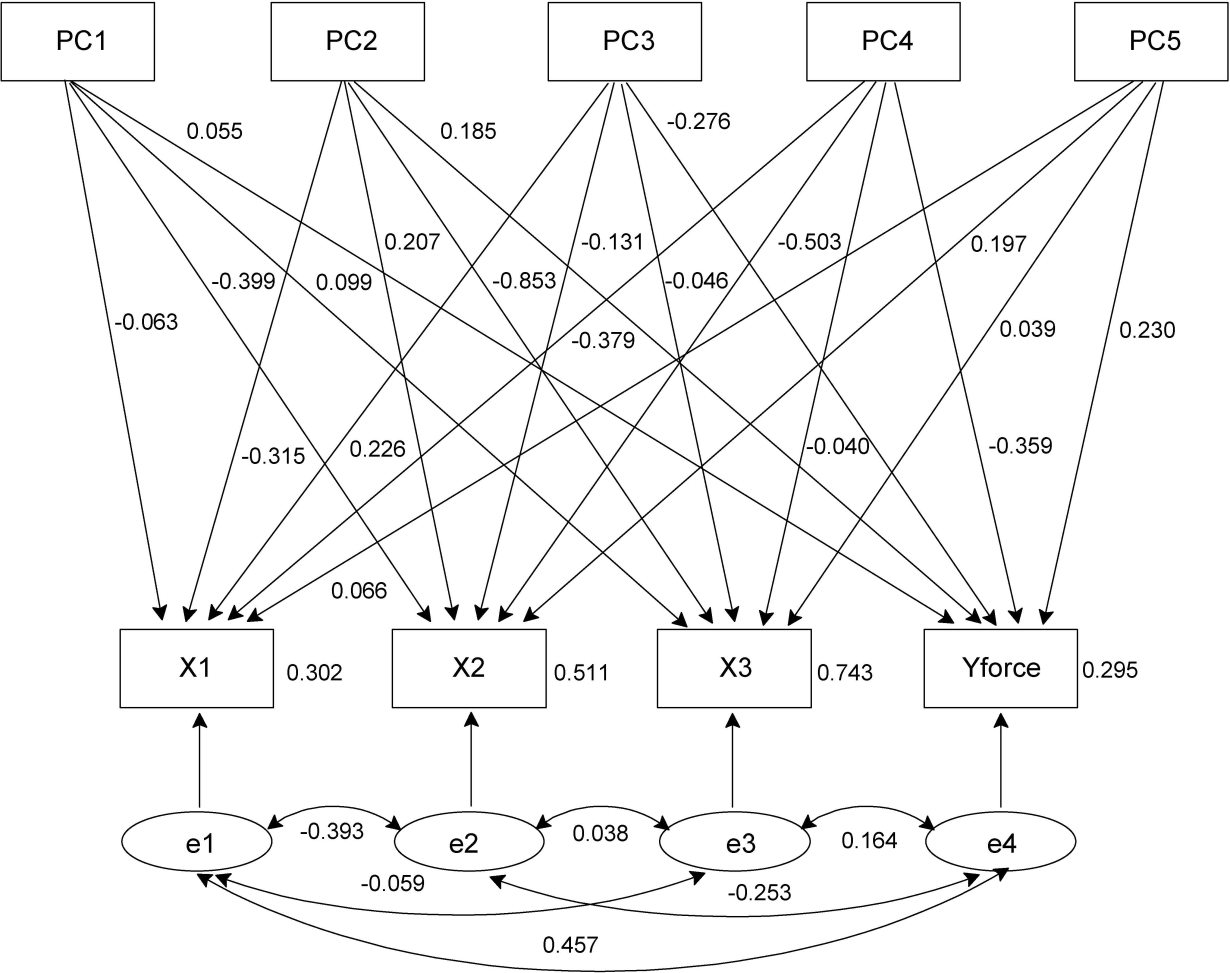
Path analysis with observed variables (Model 1).

The first five PCs (PC1-PC5) and four variables, Y_force_, X1, X2, and X3, were obtained when the maximum Y_force_ value in the tree-pulling test was used to perform the path analysis using the traditional PA-OV model. In Model 1 (**Figure 7**), the measurement indicator variables (FC1 to FC5) were obtained through PCA, and there was no correlation between them. However, a correlation between the residual terms of the variables X1, X2, X3, and Yforce was obtained from the tree-pulling experiment. The maximum absolute value of the correlation coefficient was -0.457, obtained from the correlation between X1 and Y_force_, which indicated that these two variables were related but not significantly related (P = 0.070). Based on their absolute value (influence on X1), PC1-PC5 were ranked in the order of PC4 > PC2 > PC3 > PC5 > PC1, whereas based on their effect on X2, they followed the order of PC4 > PC1 > PC2 > PC5 > PC3. On X3, however, the order of PC2 > PC1 > PC3 > PC4 > PC5 was observed, and they were in the order of PC4 > PC3 > PC5 > PC2 > PC1 based on their effect on Y_force_. The paths PC4 → X1 (P = 0.048), PC4 → X2 (P = 0.002), PC1 → X2 (P = 0.013), and PC2 →X3 (P < 0.001) showed significant differences. PC4 was the most important factor driving the stability of standing trees, followed by PC2.

**Figure 8:**
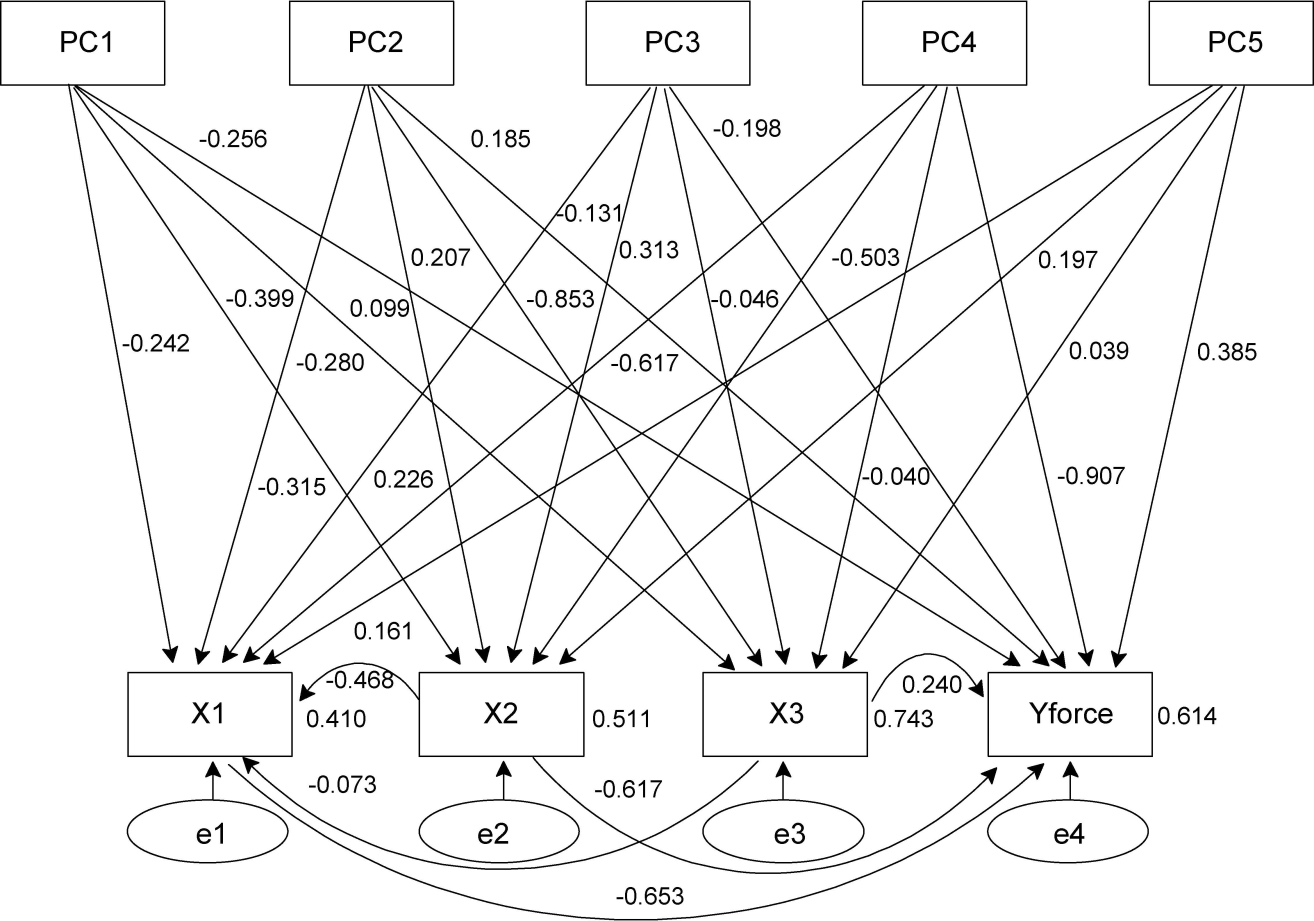
Path analysis with observed variables (Model 2).

Considering the causal relationship between the model variables, the path diagram of Model 2 was modified from that of Model 1 (**Figure 8**), which could significantly increase the explained variance of the four variables of the tree-pulling experiment. The R^2^ values of X1 and Y_force_ increased from 0.302 (model 1) to 0.410 (model 2) and from 0.295 (model 1) to 0.614 (model 2), respectively. In Model 2, based on their effect on X1, the PCs were ranked as follows: PC4 > PC1 > PC3 = PC5 > PC2, whereas based on their effect on X2, they were ranked as PC4 > PC1 > PC2 > PC5 > PC3. On X3, the order of PC2 > PC1 > PC3 > PC4 > PC5 was obtained, while based on the effect on Y_force_, the order was PC4 > PC3 > PC5 > PC1 > PC2. Therefore, PC4 was the most important factor affecting the stability of standing trees.

## 4 Discussion

The risk of wind damage to forests intensifies with the ever-changing climate (Lindner et al., 2010; Haarsma et al., 2013; Csilléry et al., 2017). To effectively reduce the risk of wind damage to plantation forests, it is necessary to identify the key factors that affect the susceptibility of trees/stands to wind damage (Nolet et al., 2012). In our study, 60 trees from 20 3-year-old *E. camaldulensis* families were selected, and their growth traits, wood density, non-destructive traits of wood, fiber morphological traits and chemical compositions, and wood properties were evaluated.

The results showed that the growth traits, wood properties, and wind damage indices of the 20 *E. camaldulensis* genotypes varied, which is consistent with the results of previous studies (Wang et al., 2014; Shang et al., 2017). The tested families showed a greater coefficient of variation, making it possible to select better genotypes with optimal growth performance and high wind resistance. Correlation analysis revealed a strong correlation between the same type of trait and a certain degree of correlation between different sets of traits. Strong correlations were observed between WD and H and between VOL and FP, which is consistent with the results of previous studies. The trunk size of trees is an important factor affecting the susceptibility of trees to wind damage (Niklas and Spatz, 2012). Tree height (H) is an important factor affecting the wind resistance of *Eucalyptus* clones (Zhu, 2006; Wang et al., 2019). However, the wind resistance of some tree species was positively correlated with H, while that of others was negatively correlated, which may be related to both the characteristics of tree species and forest age. The FP can reflect the basic density and modulus of elasticity of wood to a certain extent (Ross and Pellerin, 1991) and plays a very important role in tree resistance to wind damage (Niklas and Spatz, 2012). Both FP and WBD had the greatest effect on the wind resistance of *Casuarina* and *Acacia sinensis* in coastal shelterbelts (Wu et al., 2010). In the protected forests of Hainan Province, the higher the FP value is, the greater the elastic modulus of the wood, which contributes to the hardness of the wood and results in an optimum wind resistance performance (Hao and Cao, 2021).

The proper selection of methodology is a crucial component of this research (Davis and Cosenza, 1996; Stevens, 2002). The most critical cause of wind damage remains difficult to identify (Kamimura and Shiraishi, 2007) since the traits of standing trees are not independent of each other, and the wind resistance of trees is a complex trait. Correlation analysis revealed that the main traits affecting the wind resistance of *E. camaldulensis* were growth traits (H and V), but wood properties also play important roles in providing resistance to wind damage in trees (Putz et al., 1983; Wu et al., 2010; Xu et al., 2014; Shang et al., 2017). Hence, it is necessary to use a wide range of statistical methods to identify the driving factors. Principal component analysis (PCA) can effectively aggregate data, simplify complexity, and reduce the number of indices to a single index to compensate for the deficit of one dimension in evaluating the susceptibility of trees to wind damage (Jolliffe and Cadima, 2016). PCA of 17 traits apart from WD was further performed and yielded five new independent principal components (PC1-PC5). The static load test is currently the most advanced method for assessing tree stability. Static tree-pulling tests have been performed on open-growth urban trees to measure the force required to pull the trees to the point of failure (James et al., 2006; Peltola, 2006), evaluate the effects of root loss on the short-term stability of trees (Smiley et al., 2014), and estimate the overall stability of trees in tree risk assessments (Brudi and van Wassenaer, 2002; James et al., 2013; Sebera et al., 2014). The tree-pulling test was used to evaluate wind loads and their impacts on tree stability (Zanuncio et al., 2017; Krišāns et al., 2022), which facilitates the understanding of how various trees respond to wind. To better identify the key factors affecting the wind resistance of eucalyptus trees, we used PC1-PC5 obtained from PCA as one set of variables and X1, X2, X3, and Y_force_ obtained from the tree-pulling experiments as another set of variables for performing CCA and path analysis. The results of the effect analysis on tree-pulling variables showed that PC4, comprising HC, LC, and FW, was the main factor affecting the X1 and Y_force_ of trunk deformation and the most important complex trait affecting the stability of standing trees.

The plant cell wall is composed of cellulose, hemicellulose, lignin, polysaccharides, and proteins, which form a strong network of filaments, providing mechanical support for cells, tissues, and the whole plant (Gilbert, 2010), which can reflect the ability of the plant to resist lodging to a certain extent (Hagiwara et al., 1999). Cellulose, as the main component of the cell wall, significantly promotes the regulation of the mechanical strength of the stem, while lignin, in addition to having the same role, can determine the strength of the cell wall and the lodging resistance of the stem (Lewis and Yamamoto, 1990; Turner and Somerville, 1997). When stem/branch fracture occurs, the tensile stress caused by compression or a bending moment is greater than the resistance of the wood fiber (Gardiner et al., 2008); thus, tree trunk breakage can occur (Peltola et al., 1997). In our study, the key traits, including FW, HC, and LC, influencing wind resistance reflected the fiber morphology and cell wall composition, which is consistent with previous results corresponding to the lodging resistance of crops. An increase in cellulose content significantly improved the mechanical strength of the stem and increased the lodging resistance of wheat, rice, and soybean plants (Yang et al., 2009; Fan et al., 2012; Deng et al., 2016). Lodging resistance in crops is directly proportional to the mechanical strength of stem cell walls (Jones et al., 2001; Baucher et al., 2003), which can be improved through lignin accumulation in the cell wall (Ookawa and Ishihara, 1992; Buranov and Mazza, 2008; Li et al., 2015).

Wood fiber structure is one of the important parameters that affect wood properties. Tree species with strong wind resistance have the characteristics of high toughness, impact resistance, dense fiber, and low proportion of axial parenchyma. Researches on the physical properties of different tree species showed that wood fiber width and elastic modulus were the two most important indicators affecting the total wind damage rate, among which wood fiber width was the primary factor affecting the wind resistance ability of trees. The main impact factors vary with the wind damage grade of trees (Xu et al., 2014). The wind-resistant and weak-wind resistant strains of rubber trees have different characteristics in fiber anatomy. Wood of wind-resistant species has the characteristics of short fiber, thin wall and wide cavity, large number and uniform distribution of glial fibers (Zheng et al., 2003). The breaking rate and wind damage level of rubber trees were significantly correlated with fiber width (Zhang et al., 2020). The study results of wind damage of 50 *E. camaldulensis* families indicated that wind resistance ability was related to fiber width (Shang et al., 2017). The wind resistance of the F1 hybrid of *E. urophylla × E. grandis* was significantly correlated with FW and HC (Shen et al., 2020). The morphological characteristics of fibers varied significantly between and within trees and could be genetically controlled or changed by using different afforestation methods (Zobel and Van buijtenen, 1989). High lignin endows cell walls hardness and stiffness. The higher the mechanical compressive strength of wood, the higher the lignin content and the stronger its brittleness. On the other hand, the tensile strength, fracture strength, and impact strength will all decrease with the increase of lignin content (Pettersen, 1984; Zobel and Van buijtenen, 1989). Therefore, other wood properties changes should be pay attention to when the genetic improvement of wind resistance of Eucalyptus was carried out. Our results showed that although the trial forest suffered three typhoons at a wind speed greater than 42 m/s, *Eucalyptus camaldulensis* stands/trees that were 1-5 years old did not break or become uprooted, which is consistent with the findings of Virot et al. (2016). To minimize the loss of forests caused by wind damage, eucalyptus species or genotypes with high cellulose and lignin contents may be selected and targeted for developing forest production and management strategies in typhoon-prone areas. The findings of numerous research studies suggest that wind resistance is a result of multiple wood characteristics functioning together (Gindl et al., 2004; Borrega and Gibson, 2015; Zanuncio et al., 2017). However, further research is required to verify whether our findings are applicable to other tree species.

The primary trend in genetic improvement for eucalyptus trees encompasses the enhancement of rapid growth, high quality, and stress resistance. Eucalyptus plantations in the coastal regions of South China are susceptible to substantial losses due to typhoons. It is crucial to investigate the factors influencing the wind resistance of eucalyptus trees. In this study, the traits considered to influence wind resistance include only growth and material traits. Wind resistance in trees represents a comprehensive attribute, necessitating the consideration of factors such as site conditions, wind strength, wind duration, and planting density in practical applications. Future breeding efforts may focus on targeted genetic enhancement for wind resistance in eucalyptus trees. Additionally, corresponding control measures may be implemented in advance in future eucalyptus afforestation efforts to effectively mitigate the impact of wind damage in coastal areas.

## 5 Conclusion

Eucalyptus has high levels of heterozygosity, and many important traits, such as growth and wood properties, are quantitative and controlled by multiple genes. The wind resistance of trees is affected by many complex traits. The wind resistance of standing trees varies greatly among different varieties of eucalyptus and among different families of the same species at different ages in various environments. Therefore, in this study, a more efficient measurement technique, the tree-pulling test, was used to estimate the wind resistance of different *E. camaldulensis* families. This study revealed that the key traits affecting wind resistance were H, V, and FP via correlation analysis, while according to CCA and path analysis, the key traits were HC, LC, FW (PC4) and growth traits (PC2). Based on the results of this study, we recommend the three half-sib progenies of CA26, CA27, and CA5 for promoting better wind resistance, which is consistent with our previous studies (Shang et al., 2017). Our findings suggest that improving traits related to fiber morphology and cell wall components could enhance the wind resistance of eucalyptus plants and offer important insights into more effective management of eucalyptus plantations in coastal areas of South China.

## Data Availability

The original contributions presented in the study are included in the article/supplementary material. Further inquiries can be directed to the corresponding author.
